# Author Correction: Specification of oxytocinergic and vasopressinergic circuits in the developing mouse brain

**DOI:** 10.1038/s42003-021-02314-8

**Published:** 2021-06-17

**Authors:** María Pilar Madrigal, Sandra Jurado

**Affiliations:** Instituto de Neurociencias CSIC-UMH, San Juan de Alicante, Alicante, Spain

**Keywords:** Development of the nervous system, Neuronal development

Correction to: *Communications Biology* 10.1038/s42003-021-02110-4, published online 14 May 2021.

The original version of this Article contained an error in the author name María Pilar Madrigal, which was incorrectly given as María del Pilar Madrigal, leading to incorrect indexing. This has now been corrected in both the PDF and HTML versions of the Article.

