# Supplementary material for: Specification of oxytocinergic and vasopressinergic circuits in the developing mouse brain
Source: Commun Biol. 2021 May 14;4:586. doi: 10.1038/s42003-021-02110-4 (PMC8121848; doi:10.1038/s42003-021-02110-4)
Supplement: Supplementary file 2 — Supplementary Information [file 42003_2021_2110_MOESM2_ESM.pdf]

Supplementary Figure 1

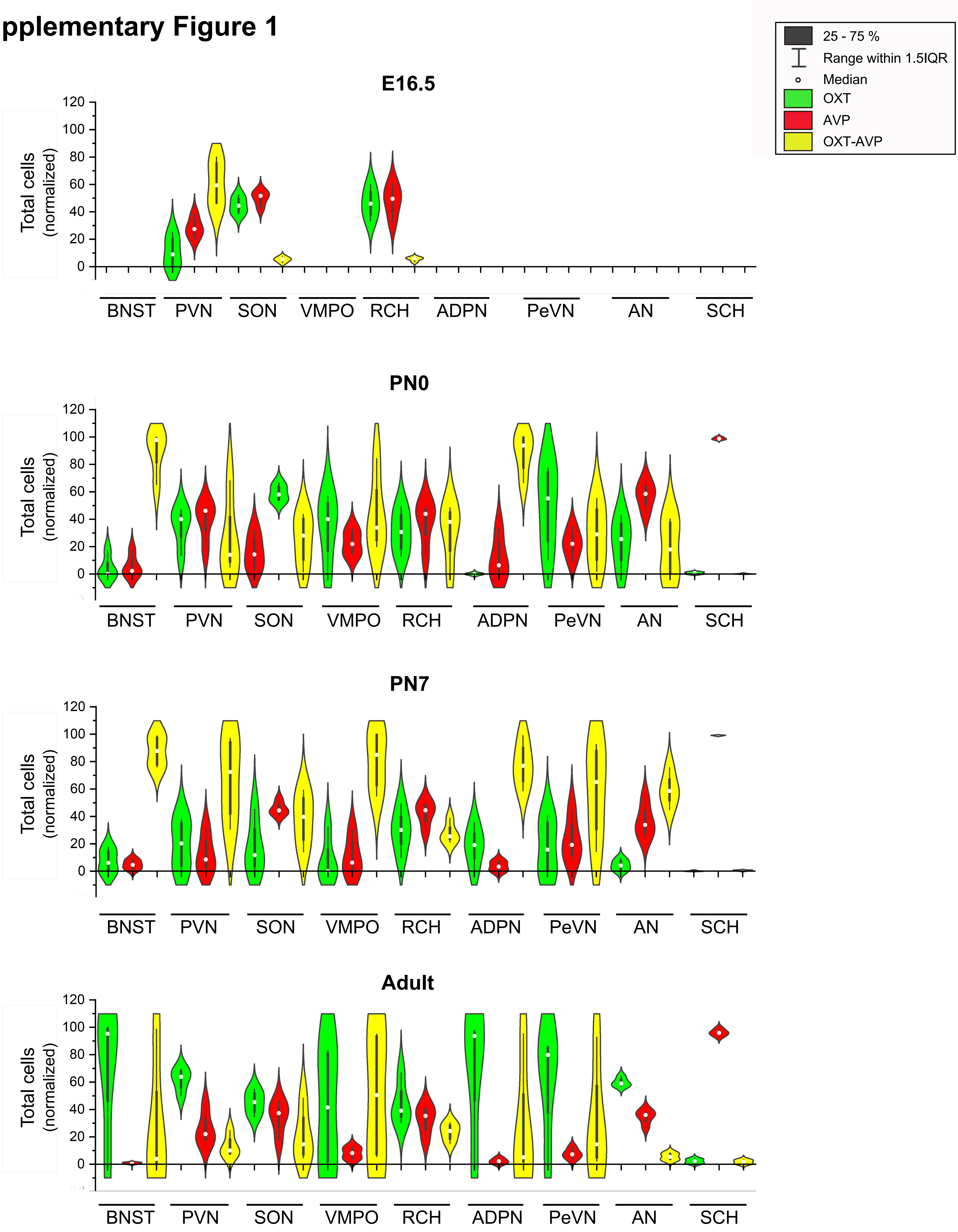

Supplementary Fig. 1 OXT and AVP dynamics during development. Violin plots represent the percentage of OXT (green), AVP (red) and OXT+AVP neurons (yellow) at E16.5, PN0, PN7 and adult. Each violin plot shows the distribution of the values as well as the median as a white dot (n=4 brains) and the interquartile range (IQR) as a thin black bar. Note that in the cases where the violin plot does not appear, it indicates that the nucleus is not yet formed.

Supplementary Figure 2

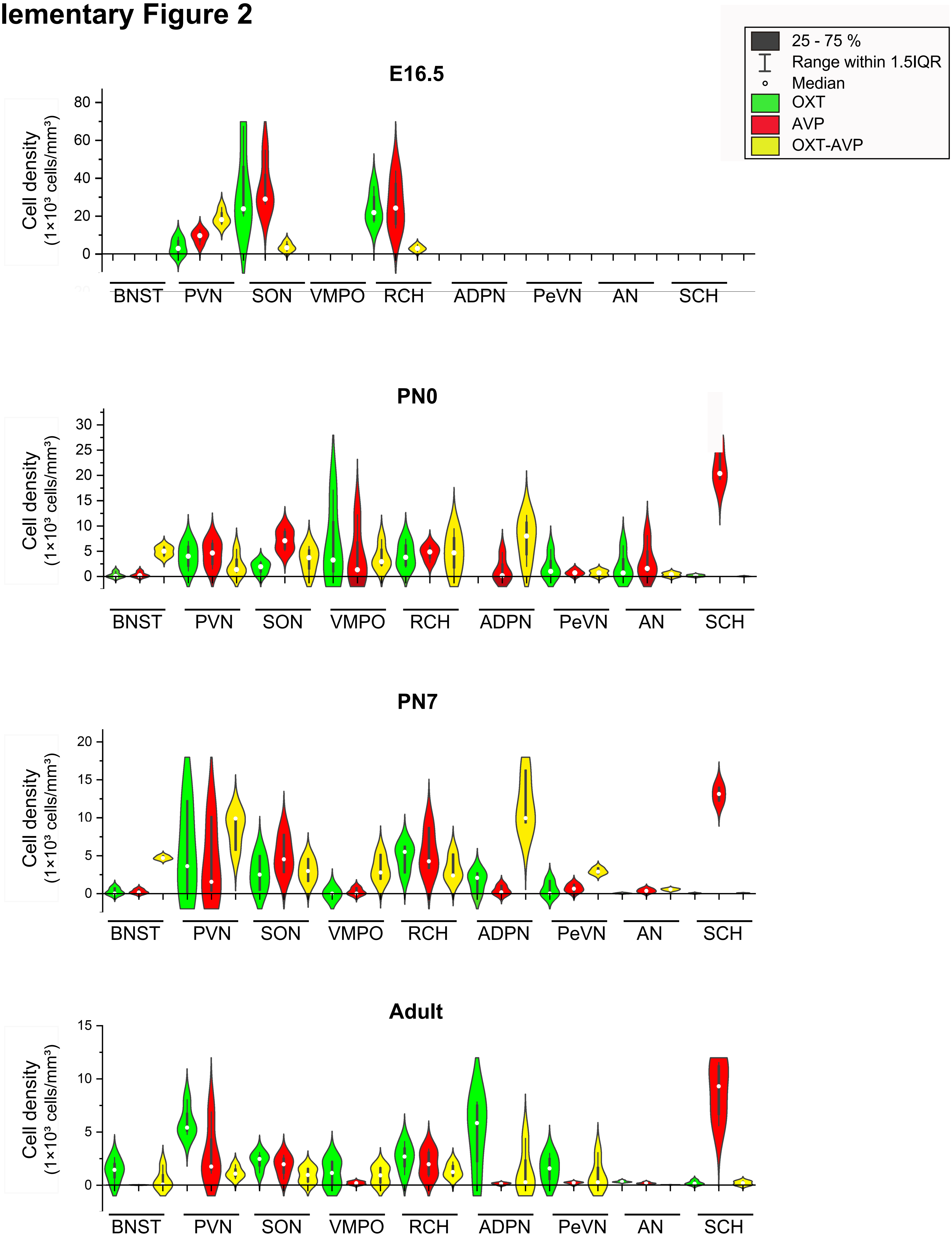

Supplementary Fig. 2 OXT and AVP expression density during development. Violin plots represent the cell density of OXT (green), AVP (red) and OXT+AVP neurons (yellow) at E16.5, PN0, PN7 and adult. Each violin plot shows the distribution of the values as well as the median as a white dot (n=4 brains) and the interquartile range (IQR) as a thin black bar. Note that in the cases where the violin plot does not appear, it indicates that the nucleus is not yet formed. Cell density is represented as 1×10^{3}cells/mm^{3}.

Supplementary figure 3

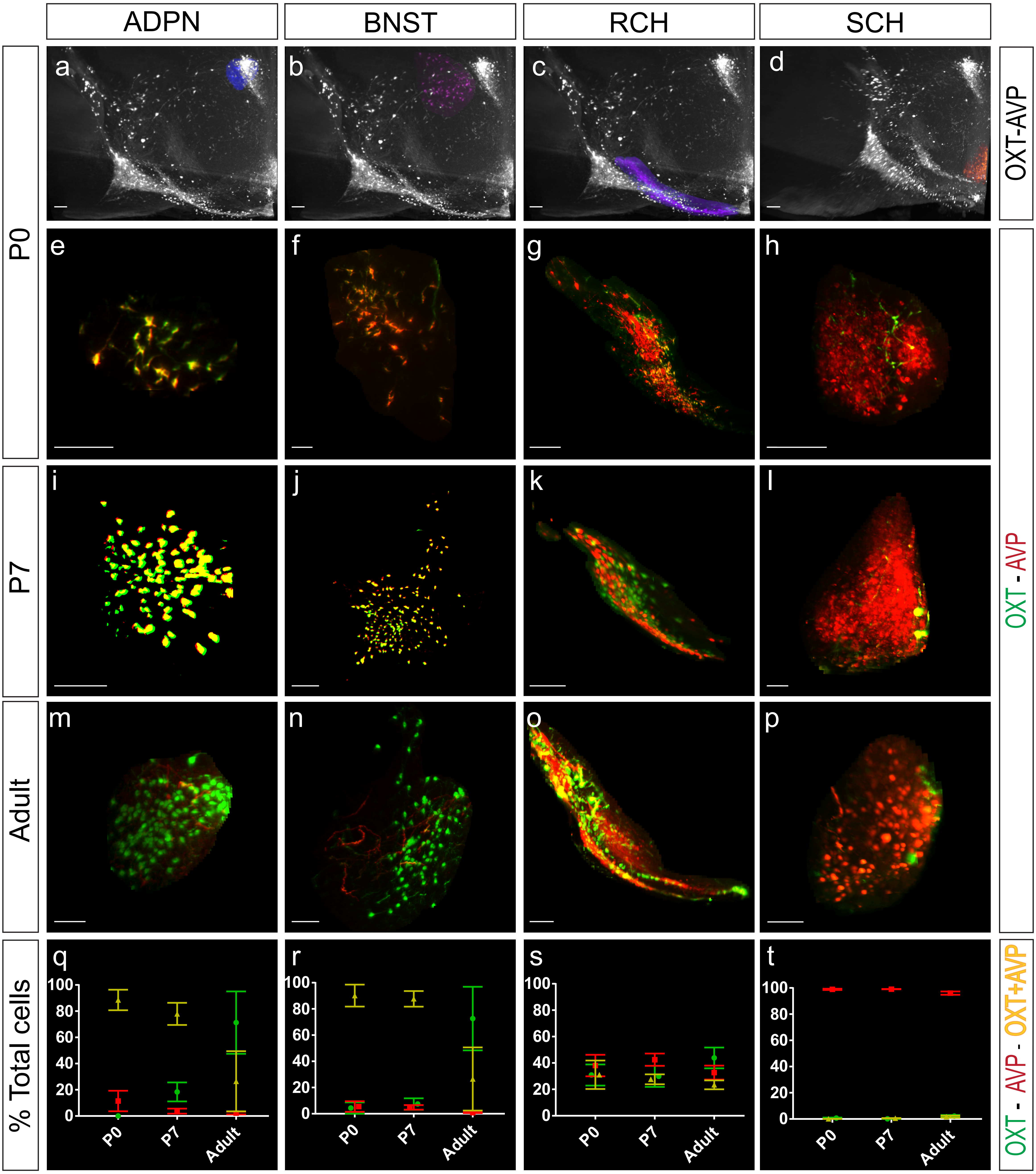

Supplementary Fig. 3 Distinct nuclei exhibit distinct OXT and AVP expression dynamics during development. Whole-mount immunolabeling against OXT and AVP at PN0 mouse hypothalamus pseudocolored in each nucleus (**a-d**). Nucleus segmentation shows OXT (green) and AVP (red) at different stages: PN0 (**e-h**), PN7 (**i-l**), and young adult (**m-p**). Percentage of each neuronal popula-tion, OXT, AVP and OXT+AVP neurons, in each nucleus during development (**q-t**; n=4 brains data are represented as mean ± S.E.M). Abbreviations: AN, accessory nucleus; VMPO, ventromedial preoptic nucleus; SCH, suprachiasmatic nucleus; RCH, retrochiasmatic nucleus. Scale bar: 100 µm.

Supplementary figure 4

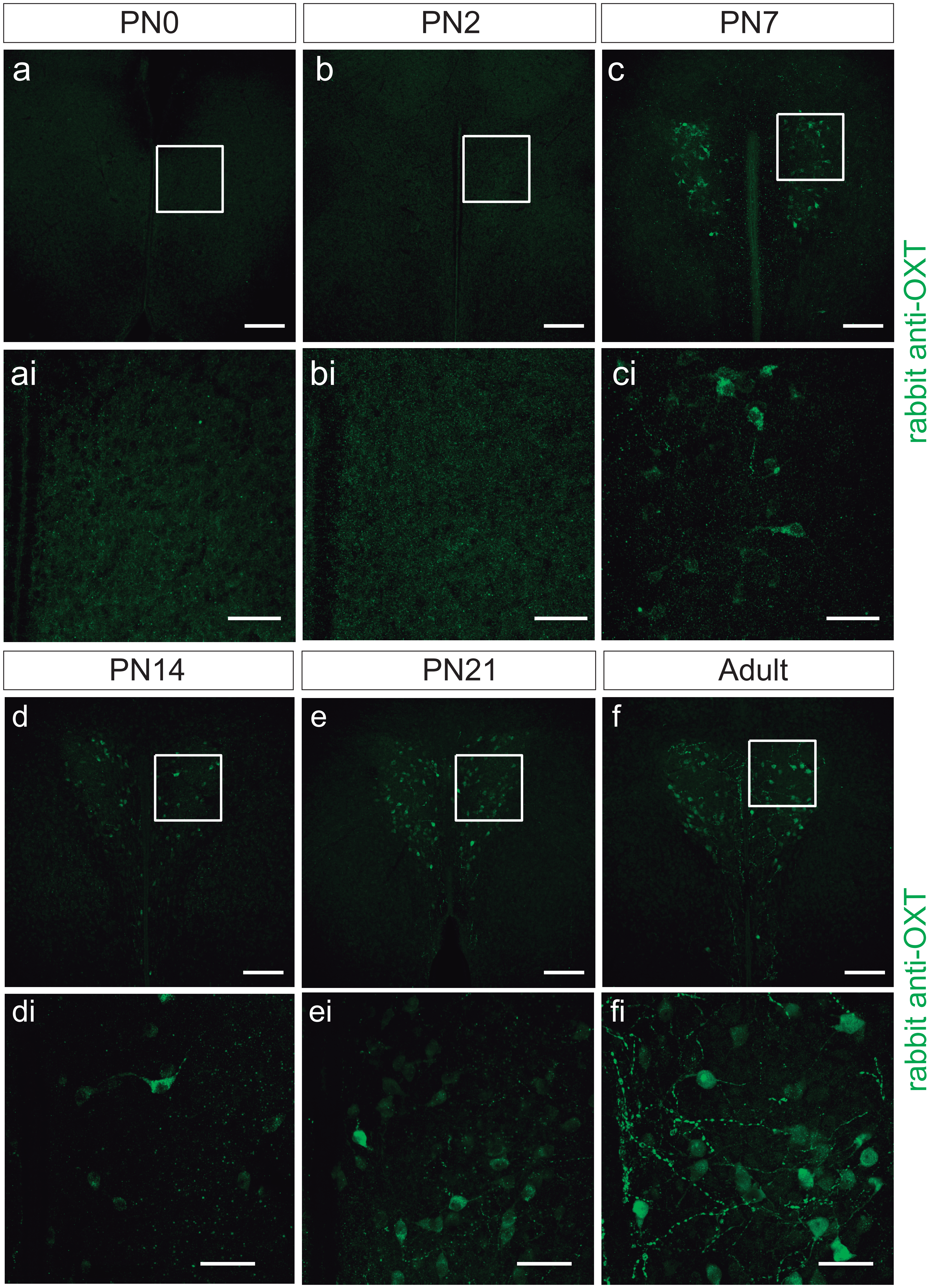

Supplementary Fig. 4 Detection of OXT expression with rabbit anti-OXT (Millipore AB911). Brain coronal sections of wild type mouse showing the PVN (**a-f**) and high magnification (**ai-fi**) at a range of developmental stages: PN0, PN2, PN7, PN14, PN21 and adult brain. Note that the initial detection of OXT with this antibody occurs at PN7. Scale bar: **a-f**: 150 μm; **ai-fi:** 50 μm.

Supplementary figure 5

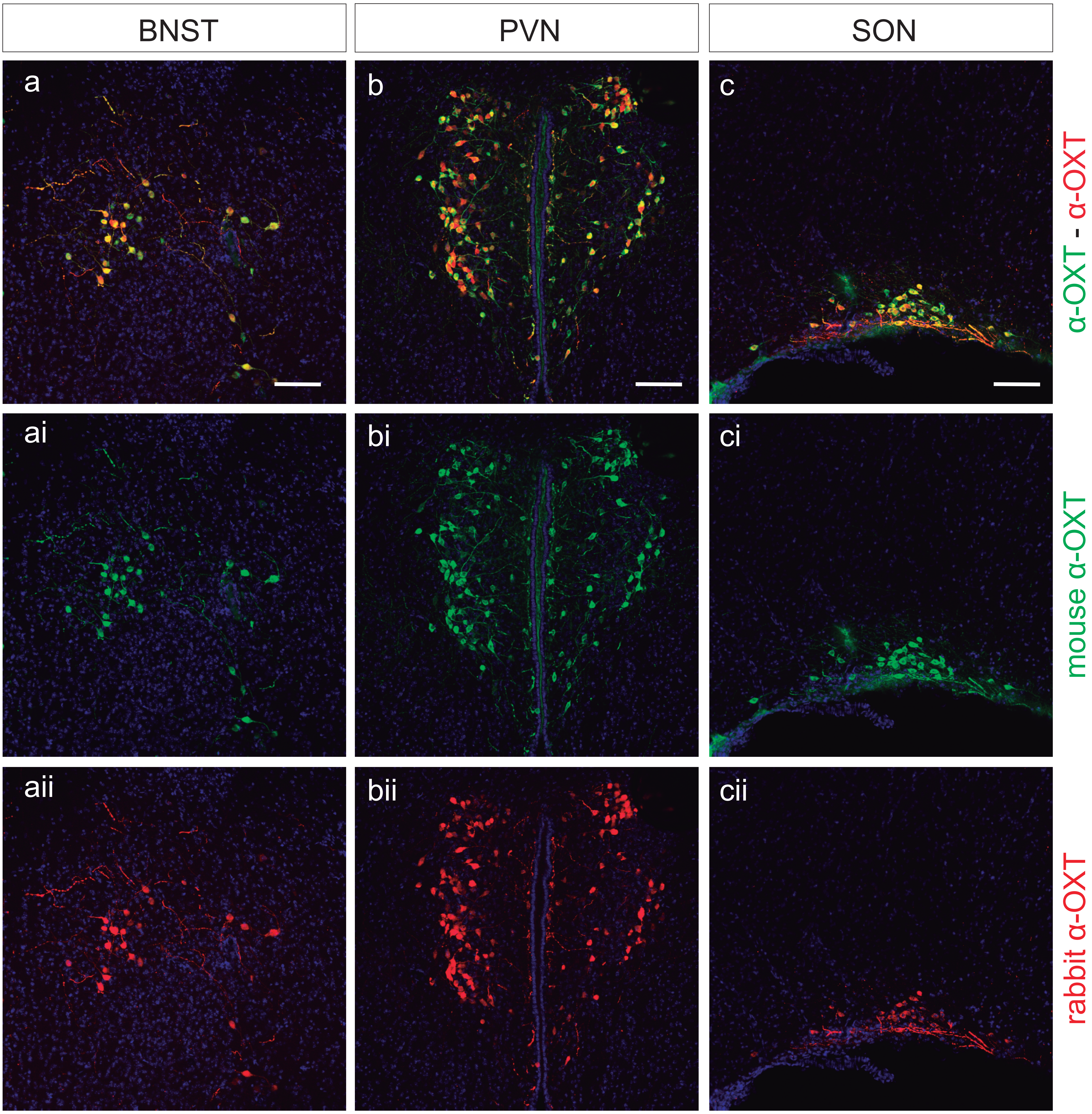

Supplementary Fig. 5 Detection of OXT expression in the adult mouse brain. Representative images of immunohistofluorescence experiments using the mouse anti-OXT PS38 and rabbit anti-OXT (Millipore AB911). Images show brain coronal sections of adult wild type mouse for a variety of regions: BNST, PVN and SON. Scale bar: 100 µm.
