# Supplementary material for: Specification of oxytocinergic and vasopressinergic circuits in the developing mouse brain
Source: Commun Biol. 2021 May 14;4:586. doi: 10.1038/s42003-021-02110-4 (PMC8121848; doi:10.1038/s42003-021-02110-4)
Supplement: Supplementary file 3 — Description of Additional Supplementary Files [file 42003_2021_2110_MOESM3_ESM.pdf]

Description of Additional Supplementary Files

File name: Supplemental Movie 1

Description: Identification of distinct OXT and AVP nuclei with iDISCO+ . Video shows a representative example of a cleared adult mouse brain. This technique allows to identify individual nuclei, which appear labeled in different colors. Abbreviations: PeVN, periventricular nucleus; ADPN, anterodorsal preoptic nucleus; BNST, bed nucleus of strial terminalis; VMPO, ventromedial preoptic nucleus; SON, supraoptic nucleus; PVN, paraventricular nucleus; RCH, retrochiasmatic nucleus; AN, accessory nucleus; SCH, suprachiasmatic nucleus.

File names: Supplementary Movies 2-5

Description: 3D reconstruction of OXT and AVP nuclei in the mouse brain over development. Whole brain immunohistofluorescence for OXT (green) and AVP (red) at different developmental stages: E16.5 (2), PN0 (3), PN7 (4) and adult (5) mouse brain. Abbreviations: PeVN, periventricular nucleus; ADPN, anterodorsal preoptic nucleus; BNST, bed nucleus of strial terminalis; VMPO, ventromedial preoptic nucleus; SON, supraoptic nucleus; PVN, paraventricular nucleus; RCH, retrochiasmatic nucleus; AN, accessory nucleus; SCH, suprachiasmatic nucleus.

File name: Supplementary Movie 6

Description: OXT neurons interconnectivity in the RCH nucleus. Video showing OXT neurons in the RCH area of an adult OXT-tdTomato mouse. Reconstruction of several coronal sections in the z axis revealed two welldifferentiated pools of OXT neurons located at the superficial and internal RCH that exhibit high levels of connectivity.
